# Imaging [^18^F]FDG PET/CT pilot study of nicotinic acetylcholinergic receptor α2 knock-out mice and α2 hypersensitive mice compared to control mice: Male-female differences and nicotine effects

**DOI:** 10.1371/journal.pone.0346469

**Published:** 2026-10-27

**Authors:** Christopher Liang, Titus E. Tucker, Ashlee D. L. Coronel, Ellen H. N. Nguyen, Janet L. Nguyen, Irakli Intskirveli, Ronit Lazar, Raju Metherate, Jogeshwar Mukherjee

**Affiliations:** 1 Preclinical Imaging, Department of Radiological Sciences, University of California, Irvine, CA, United States of America; 2 Department of Neurobiology and Behavior, University of California, Irvine, CA, United States of America; Universidade do Estado do Rio de Janeiro, BRAZIL

## Abstract

Nicotinic acetylcholinergic receptors (nAChRs), comprising of α and β subunits are present in the brain and whole body. The less abundant α2-subunit is a fast-acting receptor subtype and plays an important role in cognition and learning. To understand cellular functional consequences, this study evaluated glucose metabolism using [^18^F]FDG PET/CT in α2 knockout (α2KO) and α2 hypersensitive (α2HS) mice.

Control (CN; 4M, 3F), α2 knockout (α2KO; 4M, 4F) and α2 hypersensitive (α2HS; 4M,4F), 12–16 month old mice were used. Mice were fasted and injected with [^18^F]FDG (3–5 MBq) while awake. After 40 minutes they underwent whole body PET/CT. On a separate day, nicotine challenge [^18^F]FDG studies were done. Reconstructed images were analyzed to obtain standard uptake values (SUV) of [^18^F]FDG in brain and interscapular brown adipose tissue (IBAT). Statistical analysis was performed comparing the three groups of mice. The α2HS male mice exhibited the largest brain increase in [^18^F]FDG compared to α2KO male mice. The rank order of brain [^18^F]FDG uptake in the three groups: α2HS♂ > CN♂ > α2KO♂ > CN♀= = α2KO♀ ≥ α2HS♀. Nicotine treatment reduced brain [^18^F]FDG uptake in all mice. Females had lower [^18^F]FDG uptake compared to males and were less sensitive to α2 nAChR. In the case of IBAT, α2KO mice had significantly higher baseline [^18^F]FDG uptake compared to the other two groups: α2KO♂ > α2KO♀ > α2HS♀ > α2HS♂ > CN♀ > CN♂. Nicotine decreased IBAT in α2KO mice rather than increase as observed in CN and α2HS mice.This preliminary study shows that α2 nAChRs play a significant role in brain activation as exhibited by the increase in [^18^F]FDG in α2HS mice. In the absence of regulatory control by the α2 nAChR, the α2KO mice IBAT exhibited higher [^18^F]FDG uptake compared to controls and α2HS mice. Female mice were less affected by nicotine compared to the male mice.

## 1. Introduction

The heteromeric α4β2* nAChRs (the asterisk denotes the various subtypes including α2, α3 and α4 subunits) have a high affinity for nicotine and mediate behaviors related to nicotine addiction and thus serve as targets for nicotine cessation agents [1, 2]. Other subunits that have been implicated in nicotine-related disorders include the α2, α3, α5 and β4 nAChR subunits [3, 4]. The α2 nAChRs have attracted much attention because of their potential role in cortical function [5]. Studies in monkeys showed that the pattern of high-affinity nicotine binding better matched α2 expression than α4 expression [6]. Higher affinity of nicotine and the positron emission tomography imaging probe, [^18^F]nifene exhibited higher affinity for α2 compared to α4 nAChRs [7]. Prior studies of the α2 subunit have also shown that it plays a role in hippocampus-dependent learning and memory and potentiates several nicotine-modulated behaviors [8]. Mice with a hypersensitivity mutation of the α2 nAChR showed a deficit in fear learning which was rescued with low-dose nicotine. On the other hand, mice without the α2 nAChR exhibited learning deficits that were not rescued by nicotine administration [9]. Although the α4 nAChR has been studied more extensively than the α2 subunit, it has been shown that α2β2 nAChRs have similar responses to nicotine, positive allosteric modulators, and partial agonists of the nAChR as do α4β2 nAChRs [10]. Therefore, it appears worthwhile to study the α2 nAChR more in depth and elucidate further its role in disease models [11, 12] and nicotine modulated behaviors [13].

The habit-forming properties of nicotine acting via brain nAChR subtypes are thought to be related not only to its reinforcing properties, but also to its effects on appetite, attention, and mood [14, 15]. Acute nicotine administration has been associated with improved short and long-term memory in mice [4], whereas chronic or prenatal exposure has been shown to provoke behavior that suggests decreased attention [16, 17]. Altogether, the literature suggests that the different brain nAChR subtypes are important in a variety of cognitive pathways related to nicotine.

Nicotine has been shown to affect cerebral metabolic rates in human brains (measured using [^18^F]FDG PET), suggesting resemblance with other drugs of abuse in reducing overall brain metabolism [18, 19]. It has been suggested that nicotine enhances neurotransmission through cortico-basal ganglia-thalamic circuits directly (via nAChRs) and indirectly (via dopaminergic system) and other mechanisms [20]. This may cause differential effects of glucose metabolism in different brain regions, with increases in some brain regions while decreasing in others. Electrophysiology findings suggest that in the presence of nicotine, α2 subtype may exhibit sustained activation and may play a role in cognitive-enhancing effects of systemic nicotine [5]. Effects of nicotine on glucose metabolism via the α2 nAChR subtype remains unknown.

Glucose metabolism has also been affected by nicotine in brown adipose tissue (BAT) [21, 22]. BAT functions via thermogenesis by combusting lipids and glucose when active [23] and induced by β3 adrenergic drugs [24, 25]. Studies have shown that combinations of nAChR α3 with β2 or β4 subunits are most common in the sympathetic nervous system which controls adipose tissue activity [26]. Implicated nAChR subunits that have been found within BAT itself are α2 > β2 > β4, while in white adipose tissue (WAT) the expression is α2 > α5 > β2 [27]. The β2 subunit has been shown to play a role in nicotine-mediated expression of specific adipokines while the α2 subunit has been found to have importance in the function of beige-like adipocytes [28]. Specifically, activation of beige-like adipocytes results in induction of the α2 nAChRs in subcutaneous fat and inactivation of this receptor led to increased obesity [28, 29].

In an effort to understand the possible effects of the α2 nAChRs on glucose metabolism in the brain and BAT, we have studied three groups of mice using [^18^F]FDG PET/CT. These include C57BL/6J wild type (CN), α2 knock-out (α2KO) and α2 hypersensitive (α2HS) with both male and female mice. Baseline studies (without nicotine) and nicotine pretreatment imaging studies were carried out and compared across the three groups. Driven by the absence of α2 nAChRs or presence of α2HS nAChRs, it was hypothesized that under baseline conditions, [^18^F]FDG uptake in α2KO mice would be lower compared to CN mice, while α2HS would be higher compared to CN, in both brain and IBAT (α2HS>CN>α2KO). Nicotine-induced reduction in brain would be greatest in α2HS and [^18^F]FDG uptake would follow the rank order of α2KO>CN>α2HS and in the case of IBAT, nicotine-induced increase would follow the rank order of α2HS>CN>α2KO.

## 2. Materials and methods

### 2.1 Mice

This study was conducted under protocols approved by the University of California Irvine Institutional Animal Care and Use Committee protocol # AUP-25–068, approval period 6/13/2025–6/13/2028. Mice were housed under controlled temperatures of 70 °F, in a 12-h light–dark cycle, on at 6:00AM, with water and chow (Inotiv Teklad Global Soy Protein-Free Extruded Rodent Diet) ad libitum. For all the imaging studies mice were anesthetized with isoflurane, 3% induction, maintained at 2.5% and placed inside a Siemens Inveon PET/CT scanner. No animals were sacrificed for this work.

Table 1 summarizes the mice used in this study. Control mice (CN) were C57BL/6J strains. For α2 mice, two mouse lines with α2 nAChRs deleted (*Chrna2* -/-) for α2KO or made hypersensitive (α2HS) with a single substitution of a serine for a leucine (L9’S) (*Chrna2* L9’S/L9’S) were used. The *Chrna2* L9’S/L9’S mutation produces a 100-fold increase in sensitivity to acetylcholine in oocytes [8, 9]. The reported EC_50_ values in the presence of acetylcholine were 3.7 µM for α4β2 nAChR and for the mutant α2β2 nAChR EC_50_ was 0.041 µM [8, 9]. In rat brain slices, acetylcholine had an affinity of Ki = 19.2 nM for α4β2* nAChR’s in the presence of physostigmine (inhibitor of acetylcholinesterase) and without physostigmine the affinity was Ki = 34.7 µM [30]. Selective subtype affinity of nicotine for α2β2 Ki = 3.62 nM, α3β2 Ki = 8.46 nM and α4β2 Ki = 5.45 nM nAChR in human cloned receptors [7]. The α2HS mice would therefore be a good model to enhance functional activity mediated via the α2β2 nAChR because of this selectivity over other subtypes for acetylcholine and nicotine.

**Table 1:** Summary of mice used in the study.

Mice Type	Age, month	Weight	[^18^F]FDG MBq, ip	Brain [^18^F]FDG Scans BAT [^18^F]FDG Scans	BAT/WAT CT
***Control Mice (CN)***					
C57BL/6J (WT) Male n = 4	12 mo	34 - 46g	4.22 - 5.22 2.62 - 2.96	Baseline Nicotine, 2 mg/kg pre	Baseline Nicotine
C57BL/6J (WT) Female n = 3	12 mo	22 - 34g	3.70 - 4.51 2.96 - 4.00	Baseline Nicotine, 2 mg/kg pre	Baseline Nicotine
***α2KO Mice***					
α2KO Male n = 4	12-16 mo	18 - 60g	4.18 - 5.03 3.26 - 5.07	Baseline Nicotine, 2 mg/kg pre	Baseline Nicotine
α2KO Female n = 4	16 mo	34 - 42g	3.70 - 4.40 3.66 - 4.66	Baseline Nicotine, 2 mg/kg pre	Baseline Nicotine
***Hypersensitive α2 Mice***					
Hypersensitive α2 Male n = 4	16 mo	28 - 50g	4.12 - 4.81 4.37 - 5.03	Baseline Nicotine, 2 mg/kg pre	Baseline Nicotine
Hypersensitive α2 Female n = 4	16 mo	22 - 30g	3.92 - 4.51 3.66 - 4.22	Baseline Nicotine, 2 mg/kg pre	Baseline Nicotine

### 2.2 PET/CT imaging

Mice were fasted in the scanner room, in a dark quiet place, overnight prior to experiments based on previous procedures [31–33]. The animals were fasted for the same amount of time for baseline and nicotine scans. In the week between baseline and nicotine scans, mice weight fluctuated + /- 4 grams. [^18^F]FDG in sterile saline was purchased commercially from PETNET. On the day of the scan, all mice were placed in an enclosure maintained at 74-78^o^F. [^18^F]FDG was injected intraperitoneally with 3–5 MBq (Table 1; also see S1 Table for individual animal) in 0.01 to 0.1 mL 0.9% sterile saline, while awake without isoflurane anesthesia. Subjects were then returned to their cages and allowed to uptake the tracer in an awake, unrestrained state for 40 minutes. The subjects were then anesthetized with isoflurane, 3% induction, maintained at 2.5% and placed inside a Siemens Inveon PET/CT scanner. The PET and CT scanners were used in the “docked mode” thus enabling the acquisition of both the PET and CT scans of the mice in the same position for attenuation correction and co-registration. For all the mice, a CT scan (7 minutes) was first acquired followed by a 30-minute static PET scan. After the scan, the mice were returned to their cages. All animals recovered from the isoflurane anesthesia after the PET/CT imaging procedures. After an interval of at least a week, a second PET/CT scan on the mice was carried out, which included a co-injection of nicotine (intraperitoneally, 2 mg/kg in 0.2 mL sterile saline) along with [^18^F]FDG. Based on previous reports of IBAT mice studies, nicotine acute dose of 2 mg/kg was used.

### 2.3 Image analysis

The PET data was reconstructed as 128 (w)x128 (h)x159 (d) matrices with a transaxial pixel of 0.776 mm using an OSEM3D algorithm (2 OSEM iterations, 18 MAP iterations and 1.5 target resolution). PET images were corrected for random coincidences, attenuation and scatter using Inveon Acquisition Workplace (IAW) attenuation correction method. Calibration was conducted to Bq/cc units using a Germanium-68 phantom (cylinder 6 cm diameter), scanned in the Inveon and reconstructed under the same parameters as the mice. The CT projections were acquired with the detector-source assembly rotating over 360 degrees and 720 rotation steps. A projection bin factor of 2 was used in order to increase the signal to noise ratio in the images. The CT images were reconstructed using cone-beam reconstruction with a Shepp filter with cutoff at Nyquist frequency and binning factor of 2 resulting in an image matrix of 512x512x1008 and a voxel size of 0.052 mm. The CT images were spatially transformed and coregistered with the reconstructed PET images using the IAW software.

Brain regions and IBAT were analyzed using Inveon Research Workplace software (IRW, Siemens Medical Solutions, Knoxville, TN) using our previously described procedures [34, 35]. Brain regions such as interpeduncular nucleus (IPN), hippocampus (HIP), thalamus (TH), frontal cortex (FC), cerebellum lobes 8 and 9 (CB 8–9), and rest of the cerebellum (CB) were selected. Some of them, such as the IPN and HIP, are known to have α2 nAChR expression [8,36,37]. Activity of [^18^F]FDG was normalized using standard uptake value (SUV= ([^18^F]FDG activity in each volume of interest, VOI in kBq/mL)/ (injected dose (in kBq)/ body weight of each animal in g) methods typically used during the use of [^18^F]FDG [38]. The magnitude of BAT and WAT [^18^F]FDG was analyzed using VOIs drawn on PET images for the interscapular (IBAT) and interscapular (IWAT) region [16, 25]. Similarly to previously described methods [24 - 26] the VOIs were delineated visually using CT followed by measuring the [^18^F]FDG activity in the VOI (S1 Fig).

### 2.4 Statistical analysis

The SUV values from the PET images were analyzed in Graphpad Prism 10 and Microsoft Excel 16. For the study, 2-way ANOVA analysis with Tukey’s HSD were performed on the groups of mice (male and female CN, α2HP, α2KO with and without nicotine) with p values <0.05 indicating statistical significance. Error bars are presented as mean  ±  standard deviation. [^18^F]FDG uptake in the brain and BAT regions in the three groups of mice were compared, males and females separately. In addition, [^18^F]FDG uptake in the brain and BAT regions in the three groups of mice were compared before and after nicotine treatment.

## 3. Results

### 3.1 Control mice

Consistent with our previous findings in control mice, both male and female control mice exhibited good brain uptake under baseline conditions in ambient temperatures (Fig 1A and 1C) [39]. Thalamus (TH), interpeduncular nucleus (IPN), frontal cortex (FC), hippocampus (HP) and cerebellum (CB areas 8–9) exhibited higher levels of FDG uptake while cerebellum exhibited lower levels (Fig 1A and 1C). IBAT showed lower uptake under baseline conditions without nicotine. In the presence of nicotine, major changes occurred in brain and IBAT in both male and female mice. A significant increase in the uptake of [^18^F]FDG was seen in the IBAT along with a decrease in uptake in all regions of the brain.

**Fig 1:**
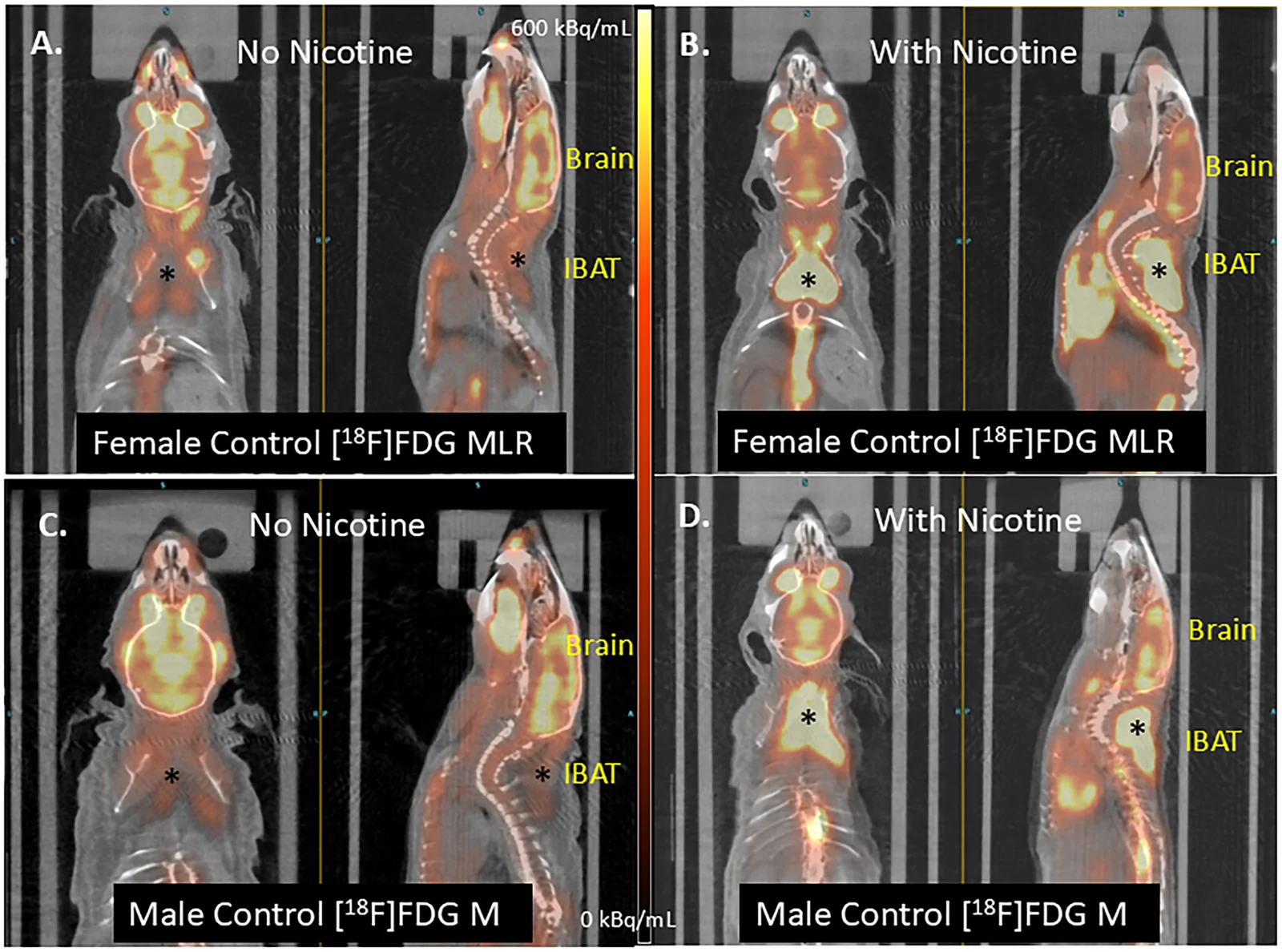
[^18^F]FDG in control mice: [^18^F]FDG uptake in C57BL/6J female mouse (A), same female mouse after pre-injection of nicotine (2 mg/kg) (B), male mouse (C), and same male mouse after pre-injection of nicotine (2 mg/kg) (D). Interscapular brown adipose tissue (IBAT) is marked by an asterisk (*). Nicotine-induced significant activation of IBAT while reducing brain uptake (B,DThe addition of nicotine induced significant activation of IBAT while reducing brain uptake (B, D).

[^18^F]FDG brain uptake ranged approximately from SUV of 3.52 to 5.37 (Fig 2A). Thalamus exhibited higher levels while CB exhibited lower levels (Fig 2A). Female mice exhibited significantly lower average brain uptake compared to male controls by approximately 20% (average females SUV = 3.89; average males SUV = 4.68; Figure 2B). Nicotine reduced [^18^F]FDG brain uptake in both male and female mice in all the brain regions with SUV levels dropping below 3.76. Both the nAChR-rich regions (TH) and nAChR-poor regions (CB) were similarly affected (Fig 2A). This global brain reduction of glucose metabolism caused by nicotine in both the male and female mice was significant (Fig 2B). Nicotine reduced the [^18^F]FDG SUV average values from 4.68 to 3.38 (28% decrease) in the male mice, while in the female mice it was reduced from 3.89 to 2.53 (35% decrease). The effect of the same dose of nicotine in male and female mice appeared to indicate a greater nicotine-induced [^18^F]FDG reduction in the female mice (Fig 2B).

**Fig 2:**
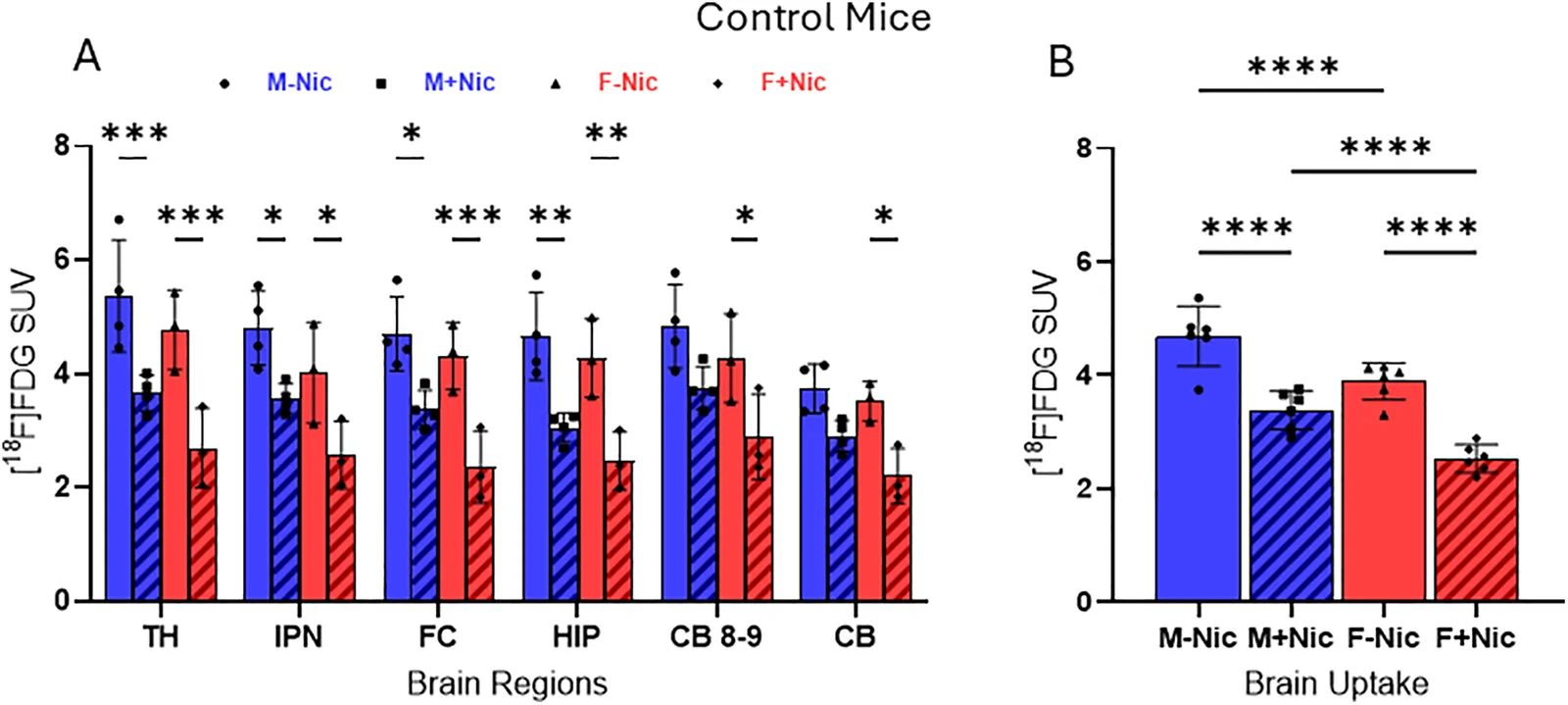
Brain distribution of [^18^F]FDG in control mice: (A). Regional brain distribution of [^18^F]FDG SUV (standard uptake value) in control C57BL/6J male and female mice before nicotine (M-Nic and F-Nic) and after nicotine (2 mg/kg, ip; M + Nic and F + Nic) administration (TH: thalamus; IPN: interpeduncular nucleus; FC: frontal cortex; HIP: hippocampus; CB 8-9: cerebellum regions 8 and 9; CB: rest of the cerebellum). (B). Whole brain [^18^F]FDG SUV average of C57BL/6J male and female mice showing significant difference (**** p < 0.0001) across sex and nicotine pretreatment. Nicotine pretreatment led to lower [^18^F]FDG SUV in both males and females. Females showed lower [^18^F]FDG SUV compared to males under both conditions.

In the same groups of mice, [^18^F]FDG uptake in IBAT and white adipose tissue (WAT) in the interscapular region were analyzed (Fig 1). Under baseline conditions (no nicotine) male mice exhibited lower IBAT uptake (SUV = 1.74) compared to the brain uptake. However, female mice exhibited significantly higher IBAT uptake (IBAT SUV = 2.88) compared to the male mice. As expected, WAT had lower levels of [^18^F]FDG uptake in both males and female mice. Contrary to nicotine treatment reducing [^18^F]FDG uptake in the brain, the uptake of [^18^F]FDG in the IBAT was dramatically increased in the IBAT of both male and female mice. Nicotine increased the IBAT [^18^F]FDG SUV values from 1.74 to 3.97 (a 228% increase) in the male mice, while in the female mice it increased from 2.88 to 6.10 (a 212% increase) (Fig 3). Although the effect of nicotine in male and female mice IBAT appeared similar, the resulting nicotine-induced [^18^F]FDG increase in the female mice IBAT was high (Fig 3). Female mice exhibited significantly higher IBAT uptake compared to nicotine treated male mice by approximately 154% (females SUV = 6.10; males SUV = 3.97; Fig 3). Thus, the sex effects on [^18^F]FDG uptake were inversed in the IBAT compared to the brain of these control mice. In the case of WAT, the effect of nicotine was statistically not significant (Fig 3).

**Fig 3:**
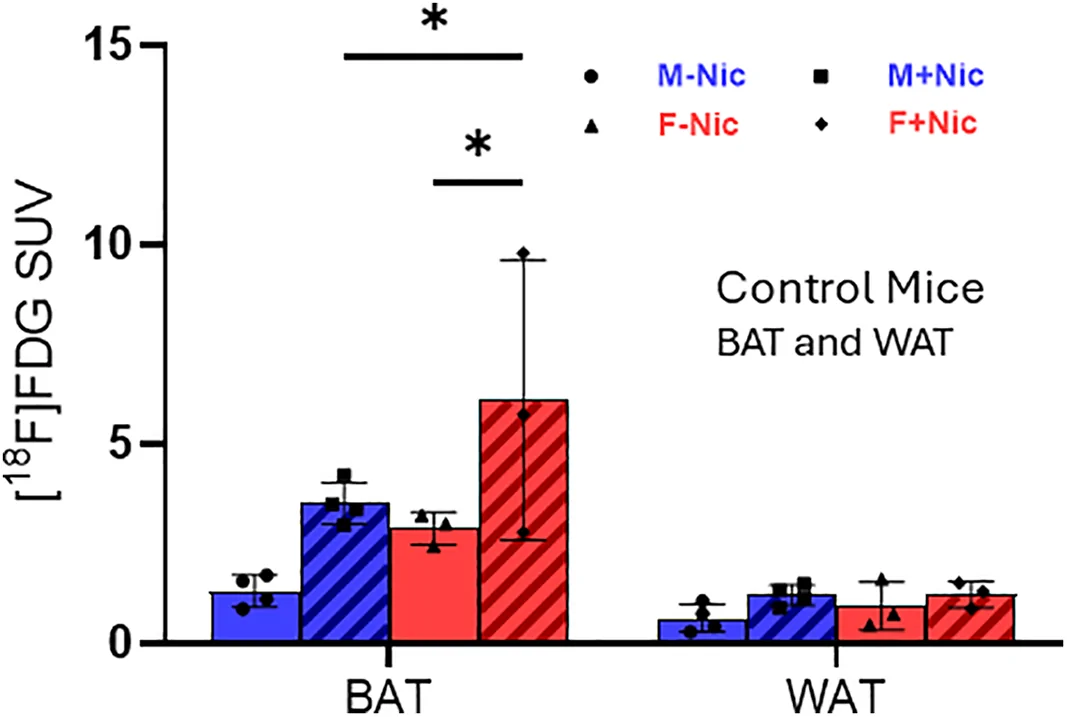
IBAT [^18^F]FDG in control mice: Brown adipose tissue (BAT) and white adipose tissue (WAT) [^18^F]FDG SUV (standard uptake value) in control C57BL/6J male and female mice before nicotine (M-Nic and F-Nic) and after nicotine (2 mg/kg, ip; M + Nic and F + Nic)Significant difference (** p < 0.01, *** p < 0.001, **** p < 0.0001, with no marking meaning no significance) is shown across sex and nicotine pretreatment. BAT uptake was higher than WAT under all conditions and nicotine pretreatment increased [^18^F]FDG SUV. Females showed higher BAT activity compared to males.

### 3.2 α2 knock-out mice

In comparison to the CN mice, major differences of [^18^F]FDG uptake in the male and female α2KO mice were observed (Fig 4). Both male and female α2KO mice exhibited good brain uptake under baseline conditions in ambient temperatures (Fig 4A and 4C). Uptake of [^18^F]FDG was seen in the TH, IPN, FC, HP and CB areas 8–9 similar to the CN mice (Fig 4A and 4C). Interscapular BAT (IBAT) showed very high uptake under baseline conditions without nicotine in both male and female mice (Fig 4A and 4C). In the presence of nicotine, major changes occurred in brain and IBAT in both male and female mice. A significant decrease in the uptake of [^18^F]FDG was seen in the IBAT along with a decrease in uptake in all regions of the brain.

**Fig 4:**
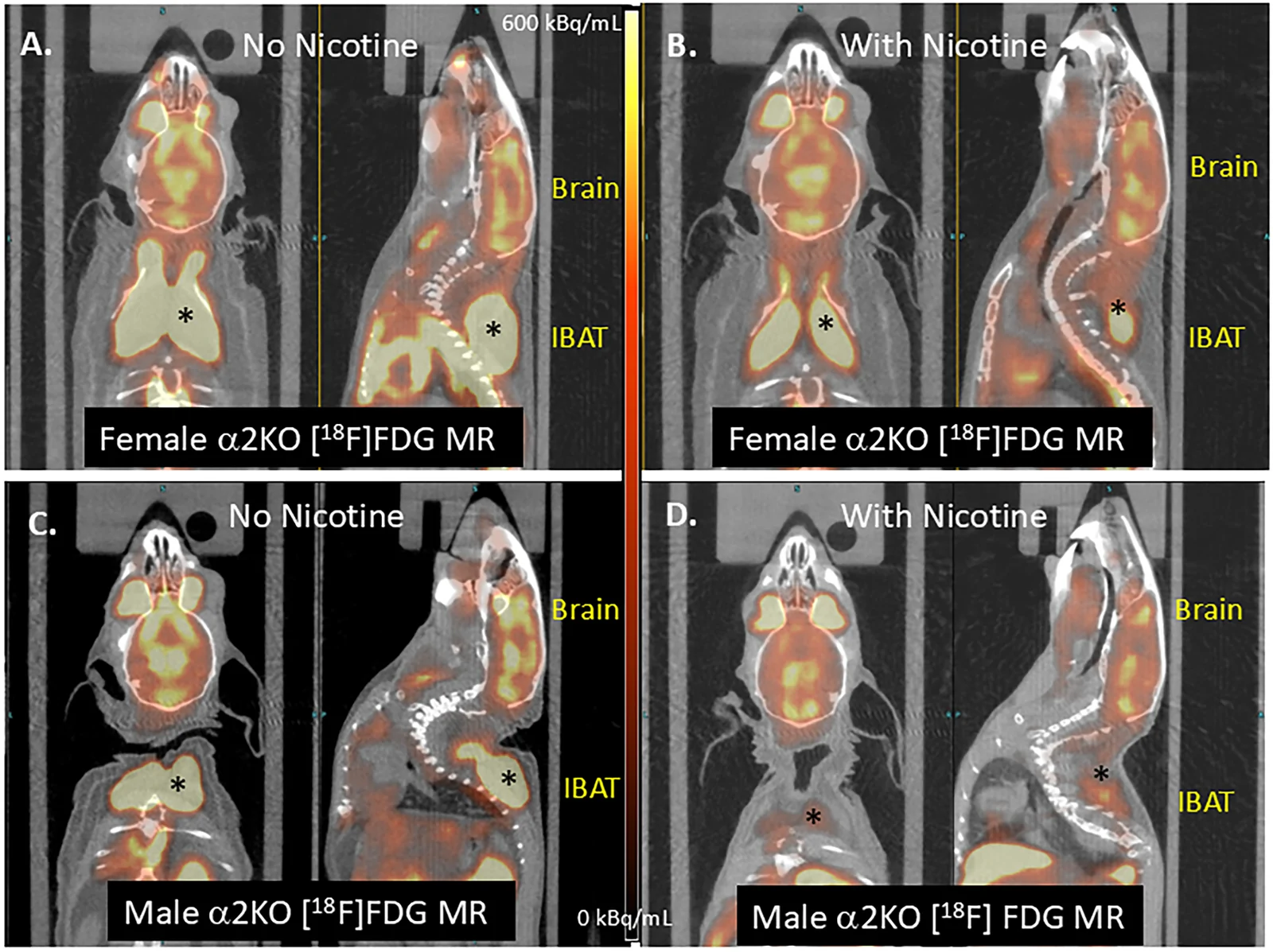
[^18^F]FDG in α2KO mice: [^18^F]FDG uptake in α2KO female mouse (A), same α2KO female mouse after pre-injection of nicotine (2 mg/kg) (B), male α2KO mouse (C), and same male α2KO mouse after pre-injection of nicotine (2 mg/kg) (D). High [^18^F]FDG uptake in the IBAT (asterisk *) is seen in baseline (A, C). Nicotine-induced significant decrease of IBAT was observed (B,D).

Brain [^18^F]FDG uptake in male and female α2KO mice ranged approximately from SUV of 3.13 to 4.44 (Fig 5A). The uptake was lower compared to the control mice. Female and male mice were similar, with lower discrimination across brain regions compared to the control mice. Female α2KO mice exhibited marginally lower average brain uptake compared to male α2KO mice by approximately 11%, but was significant (females SUV = 3.92; males SUV = 4.37; Fig 5B). Nicotine reduced [^18^F]FDG brain uptake in both male and female α2KO mice in all the brain regions with SUV levels dropping below 3.50. In female α2KO mice, the reduction in [^18^F]FDG brain uptake was less than that found in the male α2KO mice. This sex effect was seen in both the nAChR-rich regions TH and nAChR-poor regions CB (Fig 5A). The global brain reduction of glucose metabolism caused by nicotine in both the male and female mice was significant (Fig 5B) Nicotine reduced the [^18^F]FDG SUV values from 4.37 to 2.98 (a 32% decrease) in the male mice, while in the female mice it was reduced from 3.92 to 3.38 (a 14% decrease). The effect of nicotine in male and female mice appeared to indicate a greater nicotine-induced [^18^F]FDG reduction in the male mice (Fig 5B). This nicotine effect on α2KO mice was the inverse of the findings in the control mice, where the female mice exhibited a greater effect of nicotine. Thus, it appears that the α2 nAChR mediated nicotine effects may be more prominent in female mice compared to male mice.

**Fig 5:**
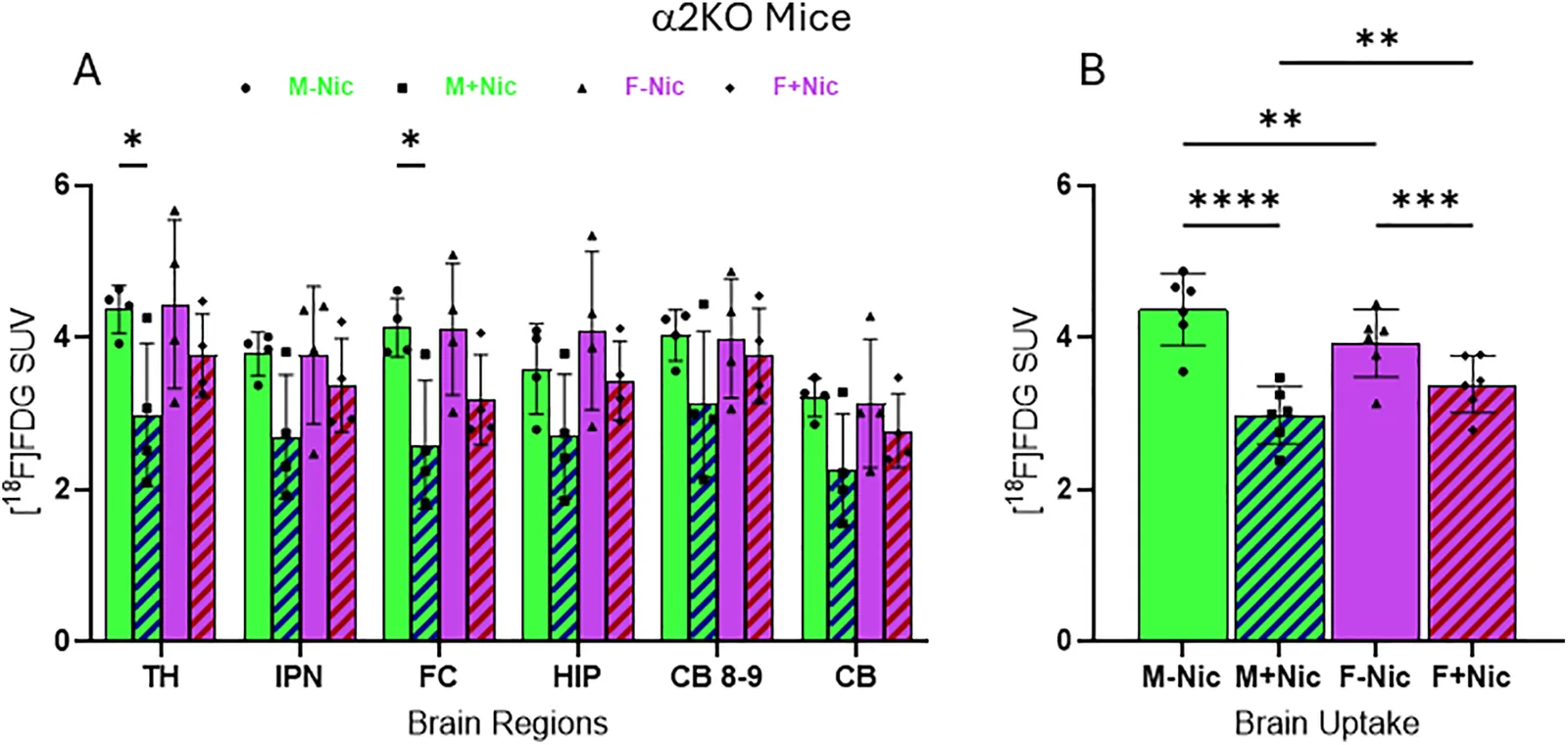
Brain distribution of [^18^F]FDG in α2KO mice: (A). Regional brain distribution of [^18^F]FDG SUV (standard uptake value) in α2KO male and female mice before nicotine (M-Nic and F-Nic) and after nicotine (2 mg/kg, ip; M + Nic and F + Nic) administration (TH: thalamus; IPN: interpeduncular nucleus; FC: frontal cortex; HIP: hippocampus; CB 8-9: cerebellum regions 8 and 9; CB: rest of the cerebellum). (B). Whole brain [^18^F]FDG SUV average of α2KO male and female mice showing significant difference (** p < 0.01, *** p < 0.001, **** p < 0.0001, ns = not significant) across sex and nicotine pretreatment. Nicotine pretreatment led to lower [^18^F]FDG SUV in both males and females. Males showed lower [^18^F]FDG SUV compared to females with nicotine pretreatment.

Analysis of [^18^F]FDG uptake in the IBAT and WAT in the α2KO mice was carried out similar to the control mice (Fig 6). Under baseline conditions (no nicotine), both male and female α2KO mice exhibited unusually high IBAT uptake (SUV = 9.05 for males; SUV = 7.86 for females) compared to the brain uptake. There was little difference between genders in both the brain and IBAT in the α2KO mice compared to the control mice. Like the control mice, WAT had lower levels of [^18^F]FDG uptake in the α2KO mice. Similar to nicotine treatment reducing [^18^F]FDG uptake in the brain, the uptake of [^18^F]FDG in the IBAT was dramatically decreased in the IBAT of both male and female α2KO mice (Fig 6). Nicotine decreased the [^18^F]FDG SUV values from 9.05 to 4.37 (a 52% decrease) in the male mice, while in the female mice it decreased from 7.86 to 4.52 (a 42% decrease). This nicotine effect in the α2KO mice was the opposite of the nicotine effect observed in the control mice. Thus, [^18^F]FDG uptake in the IBAT in the α2KO mice compared to the control mice was different in three respects: (1). A very high IBAT [^18^F]FDG uptake was observed in the baseline studies in both male and female mice; (2). Nicotine decreased IBAT [^18^F]FDG uptake in both males and females; (3). There was no significant sex effect between males and females either in the baseline or the nicotine treatment study.

**Fig 6:**
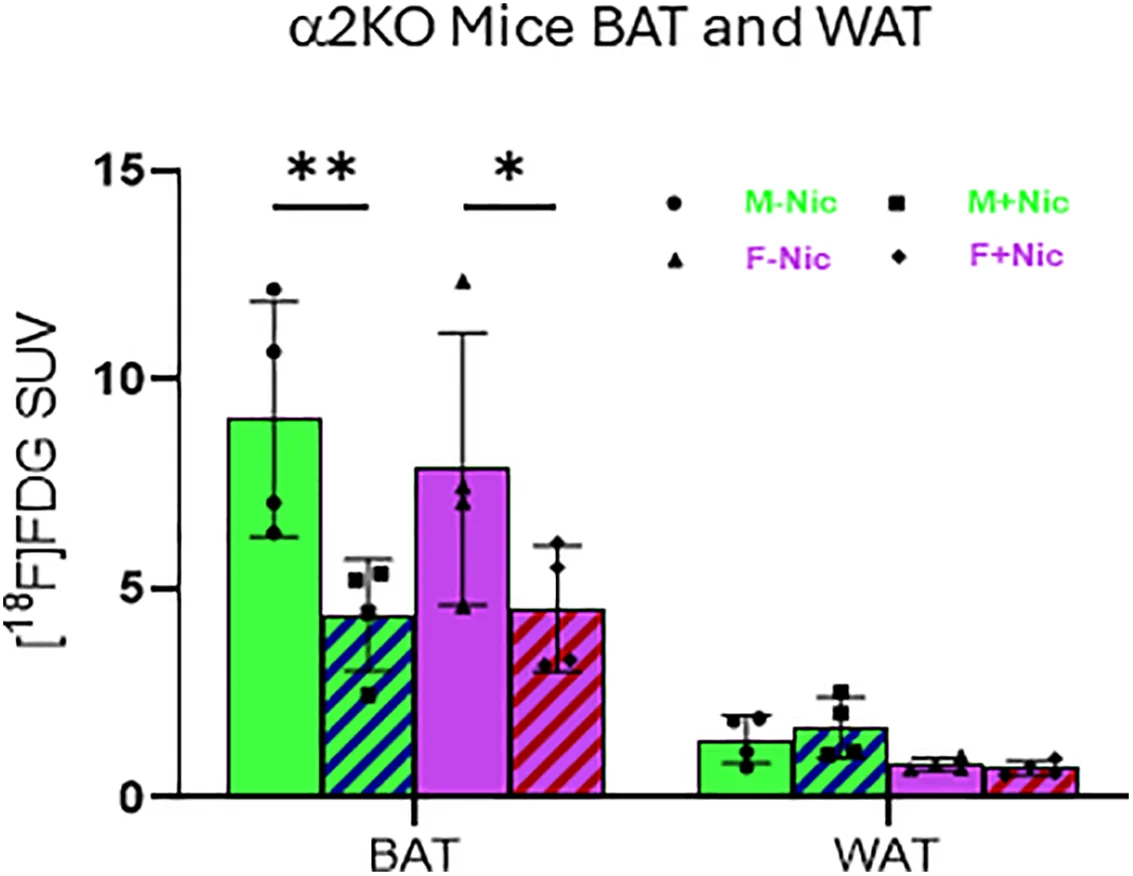
IBAT [^18^F]FDG in α2KO mice: Brown adipose tissue (BAT) and white adipose tissue (WAT) [^18^F]FDG SUV (standard uptake value) in α2KO male and female mice before nicotine (M-Nic and F-Nic) and after nicotine (2 mg/kg, ip; M + Nic and F + Nic). Significant difference (** p < 0.01, *** p < 0.001, **** p < 0.0001, with no marking meaning no significance) is shown across sex and nicotine pretreatment. BAT uptake was higher than WAT under all conditions. Nicotine pretreatment decreased [^18^F]FDG SUV in both males and females, with no significant difference between the sexes.

### 3.3 α2 hypersensitive mice

The α2HS mice exhibited [^18^F]FDG uptake more similar to the control mice (Fig 7)., Both male and female control mice exhibited good brain uptake under baseline conditions in the various brain regions. However, noticeably, male α2HS mice had significantly higher [^18^F]FDG uptake compared to the female α2HS mice (Fig 7A and 7C). IBAT showed low uptake under baseline conditions without nicotine similar to the control mice. In the presence of nicotine, a significant increase in the uptake of [^18^F]FDG was seen in the IBAT, while a decrease in uptake in all brain regions was evident.

**Fig 7:**
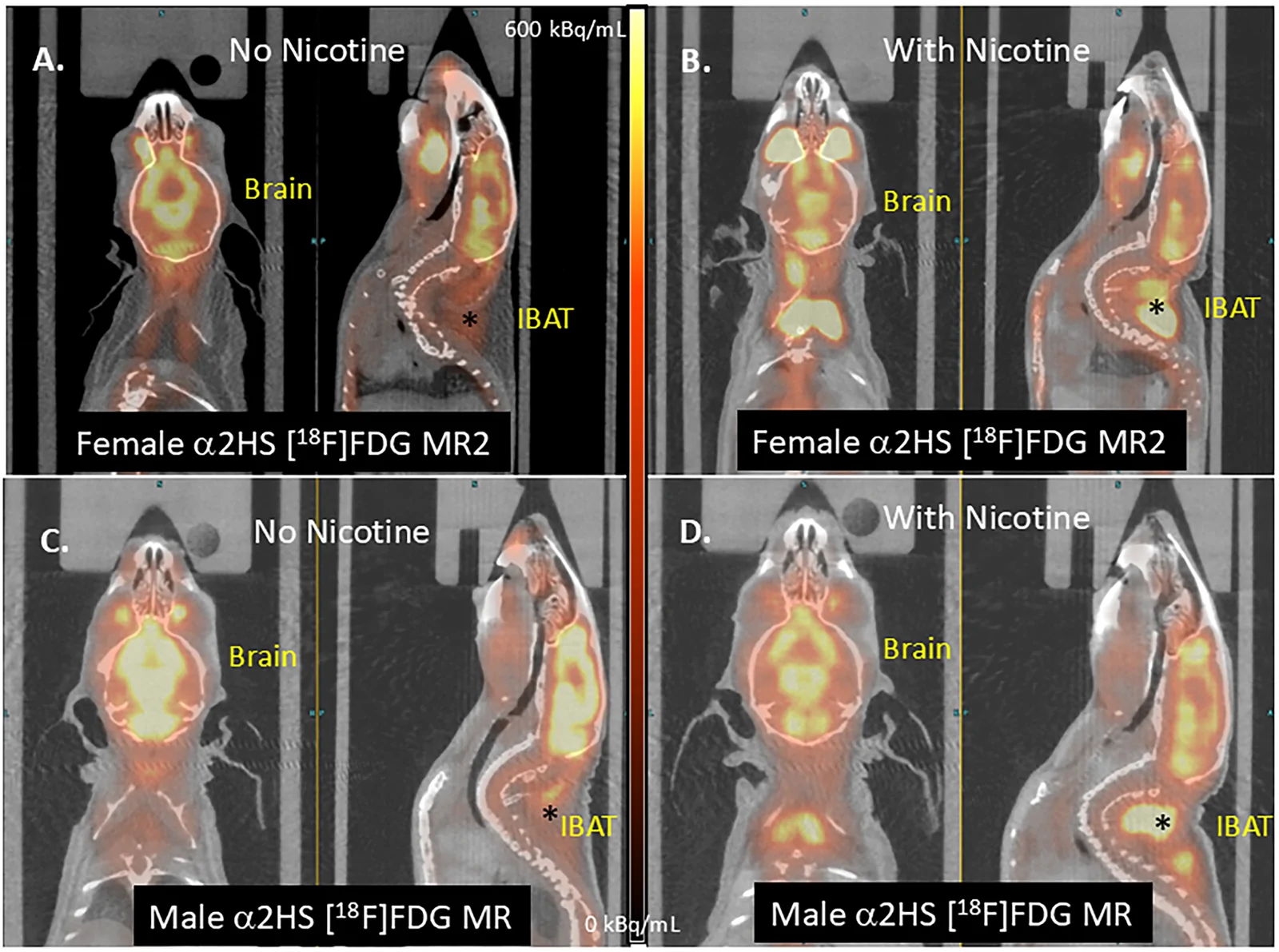
[^18^F]FDG in α2HS mice: [^18^F]FDG uptake in α2HS female mouse (A), same female α2HS mouse after pre-injection of nicotine (2 mg/kg) (B), male α2HS mouse (C), and same male α2HS mouse after pre-injection of nicotine (2 mg/kg) (D). IBAT is marked by an asterisk (*). Nicotine-induced significant activation of IBAT while reducing brain uptake (B,D).

Although both male and female α2HS mice exhibited high [^18^F]FDG brain uptake, the male α2HS mice exhibited much higher uptake (SUV=>6.00) versus female α2HS mice (SUV = 3.70 to 4.12). As in the control mice, thalamus exhibited higher levels while cerebellum exhibited lower levels (Fig 8A). Thus, of all the mice, the male α2HS mice exhibited the highest [^18^F]FDG uptake in the brain. Male-female difference in the brain was significant. Nicotine treatment caused a significant decrease in [^18^F]FDG uptake in both male and female α2HS mice. The decrease in the male α2HS mice from baseline to nicotine treatment was much larger, approximately 44% (average SUV = 5.88 to average SUV = 3.30), whereas female mice change was smaller (32%; average SUV = 3.63 to average SUV = 2.47), but significant (Fig 8B). Thus, it appears that the α2HS nAChR and nicotine may have different mechanisms/pathways that affect glucose metabolism in males and females, with females being less responsive to the increased sensitivity of the α2HS nAChR.

**Fig 8:**
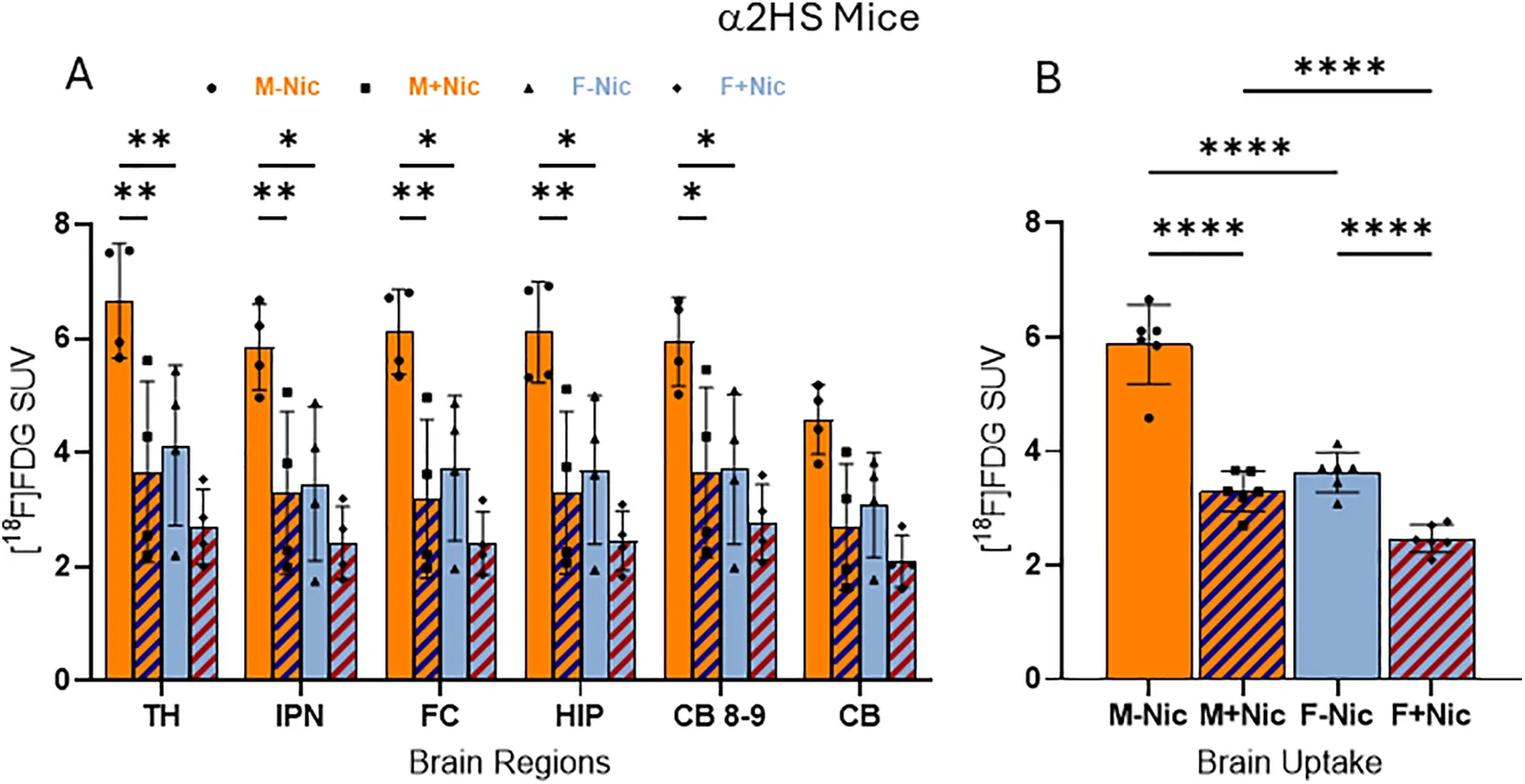
Brain distribution of [^18^F]FDG in α2HS mice: (A). Regional brain distribution of [^18^F]FDG SUV (standard uptake value) in α2HS male and female mice before nicotine (M-Nic and F-Nic) and after nicotine (2 mg/kg, ip; M + Nic and F + Nic) administration (TH: thalamus; IPN: interpeduncular nucleus; FC: frontal cortex; HIP: hippocampus; CB 8-9: cerebellum regions 8 and 9; CB: rest of the cerebellum). (B). Whole brain [^18^F]FDG SUV average of α2HS male and female mice showing significant difference (**** p < 0.0001) across sex and nicotine pretreatment. Nicotine pretreatment led to lower [^18^F]FDG SUV in both males and females. Females showed lower [^18^F]FDG SUV compared to males under both conditions. Males showed a greater decrease with nicotine pretreatment.

[^18^F]FDG uptake in α2HS IBAT and WAT in the interscapular region were similarly analyzed (Fig 9). Although the uptake profile in the two groups of mice under the two conditions were somewhat similar to the control group, there were observable differences. Under baseline conditions (no nicotine) male mice exhibited lower IBAT uptake (SUV = 3.35) compared to the brain uptake. However, female mice exhibited higher IBAT uptake (IBAT SUV = 4.04 vs brain SUV = 3.63) compared to the male mice. As expected, WAT had lower levels of [^18^F]FDG uptake. The uptake of [^18^F]FDG in the IBAT was dramatically increased in the presence of nicotine for both male and female α2HS mice. This is similar to the control mice but opposite of the α2KO group of mice. Nicotine increased the [^18^F]FDG SUV values from 3.35 to 4.79 (a 143% increase) in the male mice, while in the female mice it increased from 4.04 to 6.02 (a 149% increase). There was no decipherable effect of nicotine on WAT (Fig 9). Nicotine-induced effects appeared to be greater in the female mice (Fig 9). Thus, the sex effects on [^18^F]FDG uptake in the α2HS mice appeared to be similar to the control mice.

**Fig 9:**
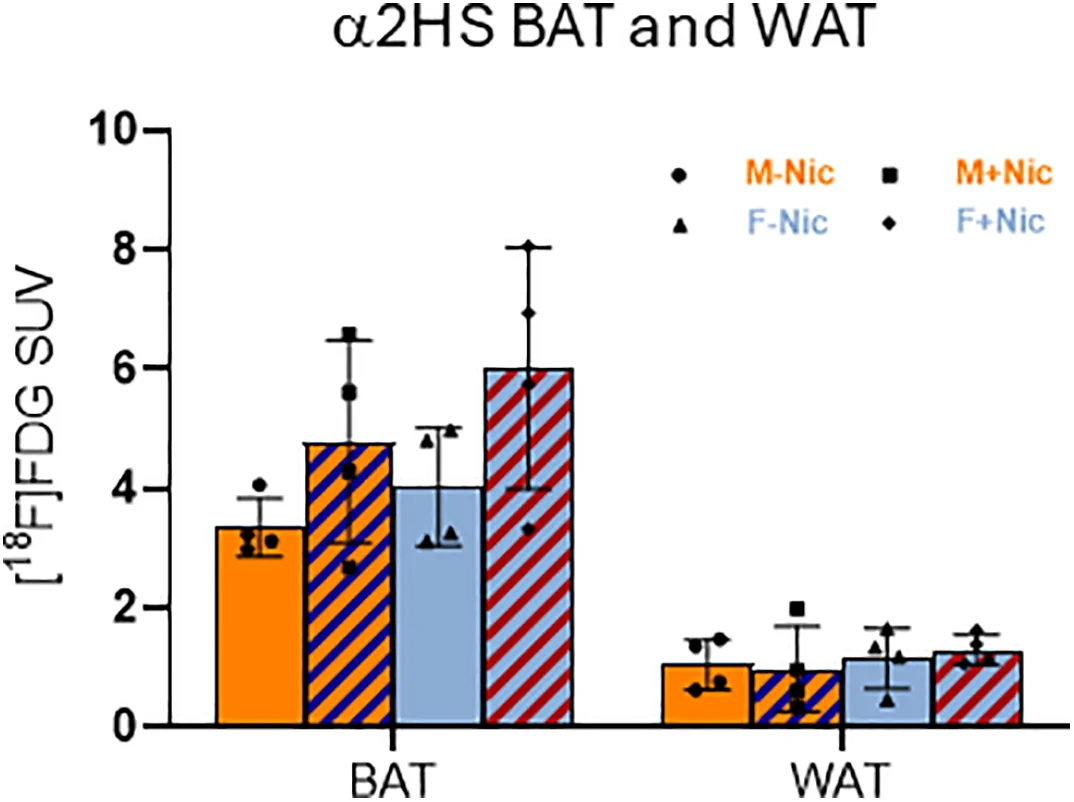
IBAT [^18^F]FDG in α2HS mice: Brown adipose tissue (BAT) and white adipose tissue (WAT) [^18^F]FDG SUV (standard uptake value) in α2HS male and female mice before nicotine (M-Nic and F-Nic) and after nicotine (2 mg/kg, ip; M + Nic and F + Nic). Significant difference (** p < 0.01, *** p < 0.001, **** p < 0.0001, with no marking meaning no significance) is shown across sex and nicotine pretreatment. BAT uptake was higher than WAT under all conditions. Nicotine pretreatment increased [^18^F]FDG SUV in both males and females, with females showing higher BAT activity.

### 3.4 Comparing brain [^18^F]FDG in control, α2KO and α2HS mice

Comparison of the whole brain [^18^F]FDG uptake in the three groups of mice is shown in Fig 10. Male α2HS mice exhibited the significantly higher uptake of [^18^F]FDG compared to control male mice while female α2HS mice did not exhibit a higher uptake compared to control female mice (Fig 10A). Nicotine decreased [^18^F]FDG uptake in all the mice and there was no difference between the α2HS and control male and female mice. Uptake of [^18^F]FDG in the α2KO mice, males and females, were lower to controls without nicotine and were statistically significant. In the presence of nicotine, there was a reduction of [^18^F]FDG. However, female α2KO mice exhibited a lower reduction compared to the male α2KO mice and the control female mice (Fig 10B).

**Fig 10:**
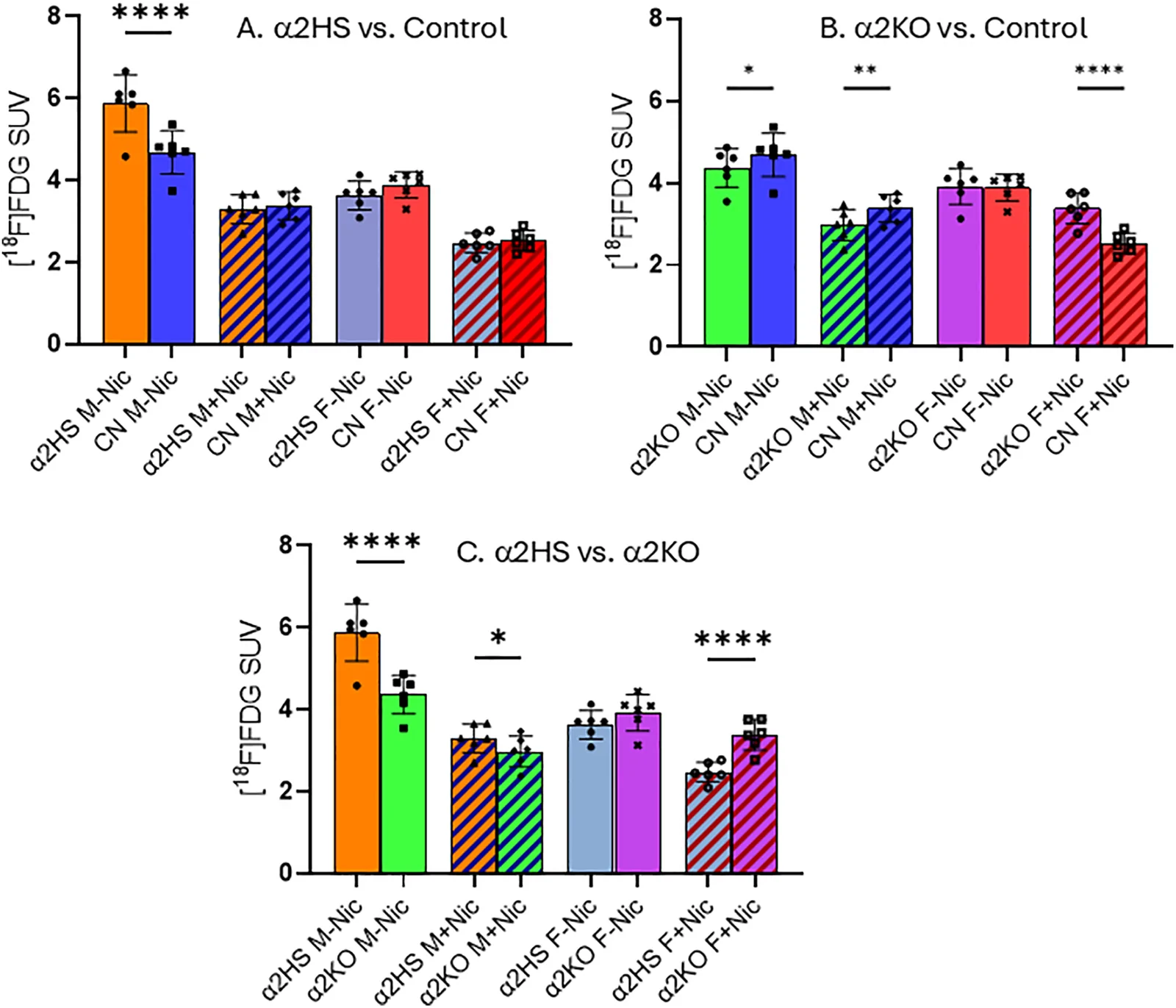
Comparison of whole brain [^18^F]FDG SUV in the three groups of mice. Brain [^18^F]FDG SUV. A. α2HS versus control C57BL/6J; B. α2KO versus control; C. α2HS versus α2KO male and female mice before nicotine and after nicotine (-NIC and +NIC; 2 mg/kg, ip). Without nicotine, α2HS male mice> α2KO male>control male mice, while female mice were similar in the three groups. Nicotine pretreatment reduced [^18^F]FDG in all mice. Males showed α2HS = controls> α2KO and females showed α2KO > α2HS = controls. (** p < 0.01, *** p < 0.001, **** p < 0.0001).

The α2HS male mice exhibited the largest difference in [^18^F]FDG compared to α2KO male mice in the absence of nicotine. The female α2HS mice did not exhibit this large a difference compared to α2KO female mice, although both males and females were statistically significant. In the presence of nicotine, there was a similar reduction of [^18^F]FDG in the male α2HS and α2KO mice. Female α2HS mice exhibited a significantly larger reduction compared to the female α2KO mice (Fig 10C). Thus, the rank order of brain [^18^F]FDG uptake in the three groups of mice was: α2HS♂ > CN♂ > α2KO♂ > CN♀ = α2KO♀ ≥ α2HS♀. In the presence of nicotine, the rank order changed to: α2HS♂ = CN♂ ≥ α2KO♂ >  = α2KO♀>  α2HS♀ = CN♀. Females had lower [^18^F]FDG uptake compared to males, with the only exception of α2KO females had higher uptake compared to α2KO males in the presence of nicotine.

### 3.5 Comparing IBAT [^18^F]FDG in control, α2KO and α2HS mice

Fig 11 shows comparison of [^18^F]FDG uptake in IBAT in the three groups of mice in the absence and presence of nicotine. The major difference was the IBAT of α2KO male and female mice compared to CN and α2HS. The α2KO mice had significantly higher baseline [^18^F]FDG uptake compared to the other two groups. More importantly, nicotine decreased IBAT [^18^F]FDG in the α2KO mice rather than the expected increases seen in the CN and α2HS mice. However, the levels of [^18^F]FDG uptake in the α2KO mice were higher compared to the baseline levels of CN and α2HS mice. Thus, the rank order of IBAT [^18^F]FDG baseline uptake in the three groups of mice was: α2KO♂ > α2KO♀> α2HS♀> α2HS♂> CN♀> CN♂. Females had higher [^18^F]FDG uptake compared to males, with the only exception of α2KO females had lower uptake compared to α2KO males at baseline. In the presence of nicotine, the rank order changed to: α2HS♀= CN♀ > α2HS♂≥ α2KO♂> = α2KO♀> CN♂.

**Fig 11:**
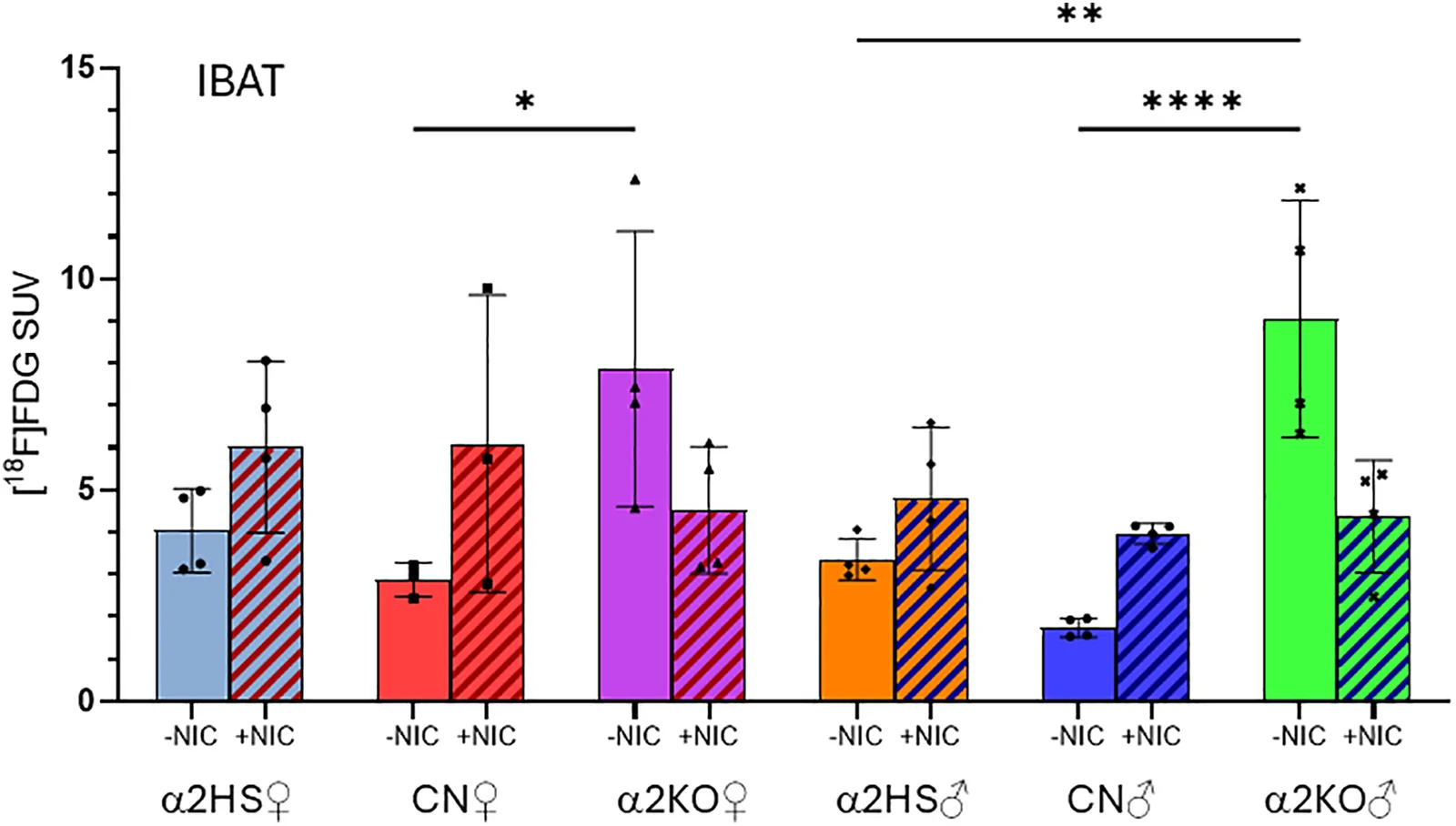
IBAT comparison of [^18^F]FDG SUV in the three groups of mice. Without nicotine, α2KO male and female mice showed higher IBAT activity compared to the α2HS and control C57BL/6J mice. Nicotine pretreatment increased [^18^F]FDG SUV in both males and females of α2HS and control C57BL/6J, but decreased IBAT activity of α2KO male and female mice. (* *p* < 0.05, ** p < 0.01, **** p < 0.0001).

## 4. Discussion

Although low in abundance, the α2 nAChRs in the human and monkey brain possess a higher affinity for nicotine compared to the other subtypes [6, 7]. These α2 nAChRs play a critical role in cortical function, mediate response to nicotine, play a role in plasticity and fear learning and memory that is modulated by nicotine [5, 9, 39]. In the sympathetic nervous system which controls adipose activity, BAT and beige-adipocytes have been shown to be abundant in α2 nAChRs [28, 29]. In this study, we have used the α2KO and α2HS mice to shed additional light on functional aspects of this receptor subtype.

[^18^F]FDG PET measures glucose metabolic activity [40], and it serves as a valuable non-invasive imaging tool for studies related to synaptic neurotransmission to study brain functional changes requiring glucose [41]. Our goal was to study the potential role of the α2 nAChR mediated effects on glucose metabolism. Previous reports in rodents [42,43] and humans have described the male-female differences in [^18^F]FDG brain uptake [44,45] and cognitive reserve with aging [46]. Studies have shown that over a 16-month period there is an age-dependent increase in [^18^F]FDG brain uptake with a positive correlation with blood glucose and weight [47] while WAT and BAT uptake does not significantly increase [48]. In the present study, male-female differences in [^18^F]FDG brain uptake were observed. Male mice showed a higher uptake compared to female mice and α2HS mice exhibited the highest brain uptake of [^18^F]FDG. Uptake of [^18^F]FDG in the α2KO mice, males and females, were lower compared to controls and were statistically significant, suggesting a role of α2 nAChR. This was further supported by the observation that α2HS male mice exhibited the largest increase in [^18^F]FDG compared to α2KO and control male mice. Surprisingly, the female α2HS mice did not exhibit such increased glucose metabolic activity compared to the control and α2KO female mice. Since control male mice exhibit higher [^18^F]FDG brain uptake compared to females, it appears that this male-female difference is further accentuated in the α2HS mice, resulting in a much greater increase in males compared to females. Overall, females exhibited less deviation in [^18^F]FDG uptake compared to males in the 3 cohorts of mice suggesting greater metabolic resistance in the females.

A number of human and nonhuman studies have used [^18^F]FDG PET to study effects of nicotine exposure on gene alterations [49] and efforts related to tobacco and smoking use [50, 51]. The action of nicotine on several nAChR subtypes directly, and indirectly on other CNS neurotransmitter-receptor systems contributes to the overall reduction in glucose metabolic activity, suggesting a decrease in synaptic neurotransmission. In this study nicotine had a similar effect of reducing glucose metabolic activity in all the three groups. The α2KO mice appeared to be less sensitive to nicotine compared to the control and α2HS mice. This effect was prominent in the α2KO female mice. The effect of nicotine was greatest in the α2HS males because of their higher baseline metabolic activity. Nicotine causes a larger decrease in metabolic in males, compared to females and this may cause the sex differences in fear conditioning in female compared to male mice [9]. The enzyme aromatase which is closely linked to estrogen levels is associated with maintaining glucose metabolism and sex hormones play a major role [52, 53]. Nicotine is a known inhibitor of aromatase [54,55] and was additionally confirmed using PET experiments in female human subjects [56]. The female α2KO mice were more resistant to the nicotine-induced reduction in glucose metabolism compared to the α2HS and control mice. This suggests that the α2 nAChR plays a significant role in nicotine effects in the female mice. Future studies could assess effects of hormone levels in these mice models in the absence and presence of nicotine.

Brown adipose tissue (BAT) is well known as a major glucose disposal tissue [46, 57]. It is known to be a major factor in energy homeostasis as well as being responsible for thermogenesis, and the adipocytes that make up the brown adipose tissue secrete factors that manage insulin, body weight, and inflammation [50, 58]. The α2 subtype is abundant within the brown and beige adipose tissue [27, 28] and has been found to have a role in increased obesity in response to high-fat diet and related metabolic disorders [28, 29]. Nicotine and tobacco can increase thermogenesis in BAT [43, 44, 50, 51]. Nicotine can induce the browning of WAT through the κ opioid receptor [51, 59] and lower WAT weight and significantly increase BAT cell count dependent on nicotine dose [52, 60].

In the absence of regulatory control by the α2 nAChR, the α2KO mice IBAT, both males and females exhibited significantly higher [^18^F]FDG IBAT uptake compared to the controls and α2HS mice. The high level of [^18^F]FDG uptake in the α2KO mice IBAT is unusual compared to the reported several other mice model studies [53, 61]. This suggests that the α2 nAChR has a complex role on IBAT glucose metabolism. Thus, non-α2 nAChR pathways may be involved in IBAT activation of α2KO mice [54, 55, 62, 63]. It is possible that the lack of α2 nAChRs during development led to a compensatory effect from other receptor systems, such as the β3-adrenergic receptor pathway or other intracellular pathways in IBAT, causing the high [^18^F]FDG uptake [25]. Future studies using inhibitors for adrenergic or nAChR systems in the α2KO mice may help in further understanding this anomalous IBAT activity. Additionally, effects of central nervous system on the sympathetic nervous system of α2KO mice may influence glucose metabolic pathways [56, 64]. At baseline (no nicotine), the hypersensitive, α2HS mice IBAT were lower compared to α2KO mice but still in the range of control mice IBAT.

In the case of nicotine treatment, male and female mice in the three groups behaved similarly with increased amount of IBAT activity compared to the brain in all groups. Nicotine induced a reduction in glucose metabolism in the α2KO mice, while in the control and α2HS mice, there was an increase in glucose metabolism which was consistent with the effects of nicotine in control IBAT [21, 22]. The nicotine-induced reduction of IBAT [^18^F]FDG in the α2KO mice was surprising and suggests a complex pathway of nicotine-induced BAT activity. The hypersensitive, α2HS mice IBAT exhibited the greatest increase in IBAT [^18^F]FDG which may be consistent with the higher amount of α2 nAChRs located in beige adipocytes, which are known to take up glucose [28, 29].

Overall, our findings showed nicotine-induced reduction in brain activity across all three groups (with a lesser effect on the α2KO mice). The α2KO mice also showed a reduction in activity in the IBAT after the addition of nicotine, suggesting that without these receptors, nicotine has the opposite relationship with these tissues. Chronic tobacco use was also significantly associated with decreased hepatic metabolic activity as measured on [^18^F]FDG-PET/CT, which suggests that smoking induces an adverse hepatotoxic effect. Nicotine abuse was also associated with decreased hepatic metabolic activity, suggesting lowered [^18^F]FDG (glucose) metabolism [42, 50]. Fig 12 shows a summary of our findings and the general trend of [^18^F]FDG distribution in the brain and IBAT across our three groups and with the addition of nicotine. α2 nAChRs have a significant effect on [^18^F]FDG uptake in the brain in male hypersensitive mice. However, for all other groups, the effect in the brain is minimal. The addition of nicotine led to a decrease in brain uptake across all three groups regardless of sex. Since dopaminergic systems degrade with age and can influence nicotine-induced changes [65,66], there is a possibility that the age of the mice was a factor. Future studies will study the effect of age in these three groups of mice. In the case of IBAT [^18^F]FDG uptake, there is a significant effect of nicotine on all three groups and both males and females, but findings of α2KO mice need further studies.

**Fig 12:**
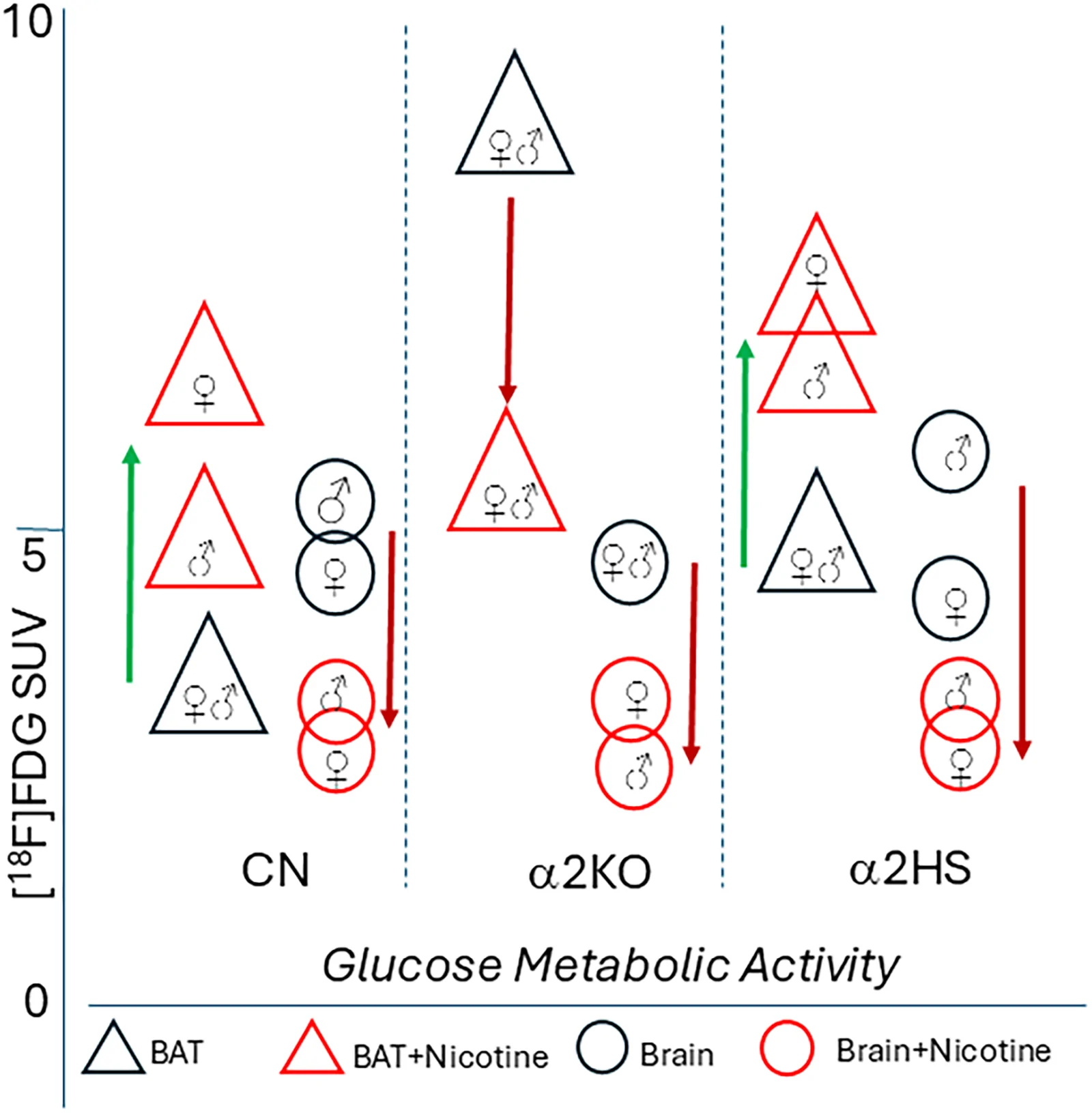
Summary of brain and BAT [^18^F]FDG SUV in the three groups of mice.

In our assessment, following are some of the limitations of this study: 1. All [^18^F]FDG imaging studies were done using the standard protocol of overnight fasting the mice to maximize brain uptake. While overnight fasting does not lead to significant changes in BAT/WAT weight in mice of our age range [67], future studies may consider non-fasted mice or a shorter fasting time to compare brain and IBAT uptake; 2. Use of lower doses of nicotine may be useful to compare the three groups and provide further information on the α2HS mice. For instance, what is the minimal amount of nicotine required to achieve a response in the α2HS model and how would it compare to control mice?3. Although significant differences amongst the three groups of animals were deciphered, increasing the number of animals would further strengthen the data and help protect against confounds. It would allow easier correction for differences in weight within the same groups of animals, allowing for extreme outliers to be accounted for; 4. The age of the mice were all 12-months or more and it will be important in future studies to see the effect of α2 modifications on younger mice. Age-related decreases in dopaminergic systems [65,66] may also affect the results, so future studies with younger mice would be done to compare nicotinic effects as well. There is also a difference in age between the control mice and the hypersensitive and knockout mice, which may affect results; 5. As a correlative study between [^18^F]FDG and α2 nicotinic acetylcholinergic receptors, more in-depth research on the precise cellular relationships and mechanisms will need to be done; 6. Acute doses of nicotine were used in this experiment and a future study examining chronic use of nicotine in knockout and hyperactive mice could further expand on the relationship between α2 nAChRs, sustained nicotine use, and brain glucose uptake. Chronic use of nicotine can lead to addiction and upregulation of nAChRs [68], which can enhance the differences between the α2KO and α2HP models. This can also open a pathway for investigating nicotine withdrawal effects and their relationship with FDG uptake. For example, α2KO mice do not display somatic manifestations of nicotine withdrawal [69], but it would be interesting to look at how the hypersensitive model would react.

## 5. Conclusions

Brain uptake of [^18^F]FDG in the α2KO mice, males and females, were lower compared to controls and higher in α2HS mice suggesting a role of α2 nAChR in glucose metabolism. Male-female differences were observed, with females having lower brain [^18^F]FDG uptake compared to males. Females were less sensitive to the presence or absence of α2 nAChR. Brain uptake in female mice was also less responsive to nicotine compared to the male mice.

[^18^F]FDG uptake in the IBAT in α2KO mice compared to the control mice was different in three respects: (1). A very high IBAT [^18^F]FDG uptake was observed in the baseline studies in both male and female α2KO mice, which was found to be contrary to our hypothesis; (2). Nicotine decreased IBAT [^18^F]FDG uptake in both males and females α2KO mice, contrary to our hypothesis; (3). There were sex differences between males and females in the baseline and the nicotine treatment study. Presence of α2HS receptors exhibited greater brain [^18^F]FDG in males compared to females. Overall, females were metabolically more resistant to alterations in α2 nAChRs compared to males.

## Supporting information

S1 Table Details of individual mice used in the study. (PDF)

S1 Fig Regions of Interest (ROI) of [^18^F]FDG in C57BL/6J female mouse PET/CT scan: (A). Coronal CT view of BAT ROI; (B). Coronal PET [^18^F]FDG uptake view of BAT ROI; (C). Sagittal CT view of BAT and WAT ROI; (D). Sagittal PET [^18^F]FDG uptake view of BAT and WAT ROI; (E). Coronal CT view of brain ROIs; (F). Coronal PET [^18^F]FDG uptake view of brain ROIs; (G). Sagittal CT view of brain ROIs; (D). Sagittal PET [^18^F]FDG uptake view of brain ROIs (Abbreviations: BAT = brown adipose tissue; WAT = white adipose tissue; TH = thalamus; CB 8–9 = cerebellar 8,9 lobes; HG = Harderian glands; FC = frontal cortex; HP = hippocampus; IPN = interpeduncular nucleus; CB = cerebellum).
(TIF)
