# Supplementary material for: Imaging [18F]FDG PET/CT pilot study of nicotinic acetylcholinergic receptor α2 knock-out mice and α2 hypersensitive mice compared to control mice: Male-female differences and nicotine effects
Source: PLoS One. 2026 Oct 27;21(10):e0346469. doi: 10.1371/journal.pone.0346469 (PMC13712757; doi:10.1371/journal.pone.0346469)
Supplement: Supplementary file 1 [file pone.0346469.s001.pdf]

Imaging [^{18}F]FDG PET/CT Pilot Study of Nicotinic Acetylcholinergic Receptor α2 Knock-Out Mice and α2 Hypersensitive Mice Compared to Control Mice: Male-Female Differences and Nicotine Effects

Christopher Liang[1], Titus E. Tucker[1¶], Ashlee D.L. Coronel[1¶], Ellen H.N. Nguyen[1¶], Janet L. Nguyen[1], Irakli Intskirveli[2], Ronit Lazar[2], Raju Metherate[2], Jogeshwar Mukherjee[1]

[1]Preclinical Imaging, Department of Radiological Sciences, [2]Neurobiology and Behavior, University of California-Irvine, Irvine, CA 92697

Emails: liangc@uci.edu (C.L.); tetucker@uci.edu (T.E.T.); adcorone@uci.edu (A.D.L.C.); honhnn@uci.edu (H.N.E.H); janetln@hs.uci.edu (J.L.N.); iintskir@uci.edu (I.I.); irlazar@uci.edu (R.L.); raju.metherate@uci.edu (R.M.); mukherjj@hs.uci.edu (J.M.)

<u>S1 Table</u>: Details of individual mice used in the study

Mice Type	Mice Name	Age, month	Weight (Baseline)	Weight (Nicotine, 2mg/kg pre)	[18F]FDG MBq, ip (Baseline)	[18F]FDG MBq, ip (Nicotine, 2mg/kg pre)
						Control Mice (CN)
C57BL/6J (WT) Male n=4	M	12 mo.	46g	44g	5.21	4.63
	ML	12 mo.	40g	34g	4.44	4.26
	MR	12 mo.	40g	36g	5.00	4.40
	MR2	12 mo.	44g	40g	4.48	4.59
C57BL/6J (WT) Female n=3	ML	12 mo.	30g	24g	4.51	4.00
	ML2	12 mo.	28g	26g	4.48	3.89
	MLR	12 mo.	32g	34g	3.70	2.96
						α2KO Mice
α2KO Male n=4	M	16 mo.	60g	56g	5.03	5.07
	MR	16 mo.	42g	38g	4.18	4.40
	MLR	16 mo.	44g	40g	4.29	5.07
	M2	12 mo.	22g	18g	5.00	3.26
α2KO Female n=4	M	16 mo.	36g	34g	4.14	4.66
	ML	16 mo.	40g	38g	4.29	3.73
	MR	16 mo.	42g	36g	4.40	3.77
	MLR	16 mo.	38g	34g	3.70	3.66
						Hypersensitive α2 Mice
Hypersensitive α2 Male n=4	M	16 mo.	48g	46g	4.81	5.03
	ML	16 mo.	50g	44g	4.81	4.55
	MR	16 mo.	28g	30g	4.11	4.85
	MLR	16 mo.	36g	36g	4.11	4.37
Hypersensitive α2 Female n=4	M	16 mo.	22g	22g	4.51	3.81
	ML	16 mo.	26g	26g	4.22	3.66
	MR	16 mo.	22g	22g	3.92	3.70
	MR2	16 mo.	30g	28g	4.37	4.22
